# Deciphering microeukaryotic–bacterial co-occurrence networks in coastal aquaculture ponds

**DOI:** 10.1007/s42995-022-00159-6

**Published:** 2023-02-22

**Authors:** Xiafei Zheng, Kui Xu, Jonathan Naoum, Yingli Lian, Bo Wu, Zhili He, Qingyun Yan

**Affiliations:** 1 Ninghai Institute of Mariculture Breeding and Seed Industry, Zhejiang Wanli University, Ningbo, 315100 China; 2 Environmental Microbiomics Research Center, School of Environmental Science and Engineering, State Key Laboratory for Biocontrol, Southern Marine Science and Engineering Guangdong Laboratory (Zhuhai), Sun Yat-Sen University, Guangzhou, 510006 China; 3 Hubei Key Laboratory of Edible Wild Plants Conservation and Utilization, Hubei Engineering Research Center of Special Wild Vegetables Breeding and Comprehensive Utilization Technology, College of Life Sciences, Hubei Normal University, Huangshi, 435002 China; 4 Department of Biological Sciences, Ecotoxicology of Aquatic Microorganisms Laboratory, GRIL-EcotoQ-TOXEN, Université Du Québec À Montréal, Succursale Centre-Ville, Montreal, QC Canada; 5 Animal Husbandry and Fisheries Research Center of Guangdong Haid Group CO., Ltd. Key Laboratory of Microecological Resources and Utilization in Breeding Industry, Ministry of Agriculture and Rural Affairs, Guangzhou, 510006 China

**Keywords:** Microeukaryote, Bipartite network, Interactions, Keystone taxa, Nestedness

## Abstract

Microeukaryotes and bacteria are key drivers of primary productivity and nutrient cycling in aquaculture ecosystems. Although their diversity and composition have been widely investigated in aquaculture systems, the co-occurrence bipartite network between microeukaryotes and bacteria remains poorly understood. This study used the bipartite network analysis of high-throughput sequencing datasets to detect the co-occurrence relationships between microeukaryotes and bacteria in water and sediment from coastal aquaculture ponds. Chlorophyta and fungi were dominant phyla in the microeukaryotic–bacterial bipartite networks in water and sediment, respectively. Chlorophyta also had overrepresented links with bacteria in water. Most microeukaryotes and bacteria were classified as generalists, and tended to have symmetric positive and negative links with bacteria in both water and sediment. However, some microeukaryotes with high density of links showed asymmetric links with bacteria in water. Modularity detection in the bipartite network indicated that four microeukaryotes and twelve uncultured bacteria might be potential keystone taxa among the module connections. Moreover, the microeukaryotic–bacterial bipartite network in sediment harbored significantly more nestedness than that in water. The loss of microeukaryotes and generalists will more likely lead to the collapse of positive co-occurrence relationships between microeukaryotes and bacteria in both water and sediment. This study unveils the topology, dominant taxa, keystone species, and robustness in the microeukaryotic–bacterial bipartite networks in coastal aquaculture ecosystems. These species herein can be applied for further management of ecological services, and such knowledge may also be very useful for the regulation of other eutrophic ecosystems.

**Supplementary Information:**

The online version contains supplementary material available at 10.1007/s42995-022-00159-6.

## Introduction

In aquatic ecosystems, microeukaryotes play critical roles as primary producers, predators, decomposers and parasites, whereas bacteria are the major drivers of nutrient cycling (de Vargas et al. 2015; Karlusich et al. 2020; Louca et al. 2016). Their inter-kingdom interactions are considered the basis of nutrient fluxes, energy flow, biogeochemical cycles, and community assembly (Zhang et al. 2020). Current studies on microeukaryotic–bacterial interactions are primarily based on culturing and the addition or removal of some particular species (Bjorbækmo et al. 2020), an approach that has largely restricted our understanding of the more than 99% of the unculturable microorganisms. In addition, previous studies typically involved one-mode networks to describe co-occurrence relationships in microbial communities, in which the microbes (nodes) can connect to each other and may include both intraspecies and interspecies relationships (Li et al. 2020; Mikhailov et al. 2019). Thus, they do not specifically focus on the relationships between different types of microbes, such as those between microeukaryotes and bacteria. However, bipartite networks (two-mode networks) can provide a systematic way of representing data that consists of two distinct guilds, such as plant-pollinator, parasite–host, or predator–prey (Burkle et al. 2013; Ferrari et al. 2011; Morris et al. 2014). For example, Bjorbækmo et al. (2020) studied the ecological interactions among aquatic protists and other microbes using bipartite networks. However, these microbial interactions were mainly based on light microscopy observations, which cannot fully reflect the interactions between microeukaryotes and bacteria. Fortunately, high-throughput sequencing of microbiomes has enabled the study of co-occurrence networks with greater depth and accuracy (Faust and Raes 2012). Thus, there are now new opportunities to understand potential cross-kingdom interactions by using bipartite network analysis with metagenome-based sequencing data.

Deciphering microeukaryotic–bacterial bipartite networks will lead to a better understanding of cross-kingdom microbial interactions, which are generally classified as positive or negative relationships. Positive interactions include symbiosis, mutualism and commensalism, while negative interactions include parasitism, amensalism and predation (Faust and Raes 2012). In freshwater ecosystems, algal–bacterial interactions are mainly positive (Seymour et al. 2017), whereas bacteria grazed by protists (e.g., ciliates and flagellates) are mainly negative interactions (Pernthaler 2005). Some microeukaryotes or bacteria in aquatic ecosystems may be involved into both positive and negative interactions (Gao et al. 2019; Muhlenbruch et al. 2018). Unfortunately, the microeukaryotic–bacterial interactions in water and sediment are poorly understood. Moreover, protists and bacteria can be classified as either generalists (holding many interactions) or specialists (holding few interactions) according to the links identified (Dormann 2011). Generally, protist predators tend to be classified as generalists while parasitic and symbiotic interactions are often classified as specialists (Simmons et al. 2019). Both generalists and specialists in nested patterns tend to interact with generalists, whereas specialist-to-specialist interactions are uncommon (Mariani et al. 2019). Typically, generalists tend to have profound effects on the network stability as the elimination of highly connected species may lead to further extinctions, ultimately affecting the entire network (Palacio et al. 2016). Therefore, identifying potential keystone taxa that may affect the microbial communities is especially important to understand the microeukaryotic–bacterial interactions.

Microeukaryotic–bacterial interactions have attracted great attention for their biotechnological applications in wastewater treatment (Abinandan and Shanthakumar 2015), biofuel production (Zhang et al. 2020), agriculture (Qiu et al. 2019), and aquaculture (Natrah et al. 2014). Neglecting microbial interactions can lead to a failure in such applications or result in unforeseen consequences. For example, a supplement of microbial consortia facilitated the growth of cyanobacteria in aquaculture ponds (Zheng et al. 2017a). Some bacteria are also able to inhibit algal growth, which can be used to terminate the harmful algal blooms (Wang et al. 2010). Considering not all microbial species contribute equally to the stability of aquatic ecosystems, study of some keystone microbial taxa may help to manage the aquatic ecosystem by optimizing their potential bio- and eco-functions (Liu et al. 2022). Consequently, there is an urgent need to better understand microeukaryotic–bacterial interactions of the potential keystone taxa (Ramanan et al. 2016).

Aquaculture continues to be the world’s fastest growing food production sector and has already exceeded global capture fisheries (Tacon 2020). Coastal ponds are typical aquaculture ecosystems which have expanded rapidly (Ren et al. 2019). However, there is almost no understanding of microeukaryotic–bacterial co-occurrence bipartite networks in coastal aquaculture ecosystems. Microbial community diversity and environment physiochemical parameters change dramatically during the aquaculture process (Zhang et al. 2016). Some microorganisms can leave the active interactions networks by entering a dormant state when the environment is not ideal (Lennon and Jones 2011). However, it is not clear how the loss of particular bacteria or microeukaryotes will influence the stability of the microeukaryote-bacteria co-occurrence relationships. We hypothesized that there are some keystone taxa that regulate the microeukaryotic–bacterial co-occurrence networks in water and sediment, and that the loss of generalists and microeukaryotes may more easily lead to a collapse of microeukaryotic–bacterial bipartite networks. This study used bipartite network analysis to explore potential microeukaryotic–bacterial interactions in coastal carp aquaculture ponds. Our aim were to: (1) determine the co-occurrence bipartite network topology between microeukaryotes and bacteria; (2) identify the core and keystone taxa affecting the microeukaryotic–bacterial bipartite network; and (3) clarify the stability of microeukaryotic–bacterial relationships in water and sediment. This study provides fundamental knowledge for microbial cross-kingdom relationships, which will guide manipulations of microbial remediations in coastal aquaculture and other aquatic ecosystems.

## Results

### Overview of the bipartite network between microeukaryotes and bacteria

**Fig. 1:**
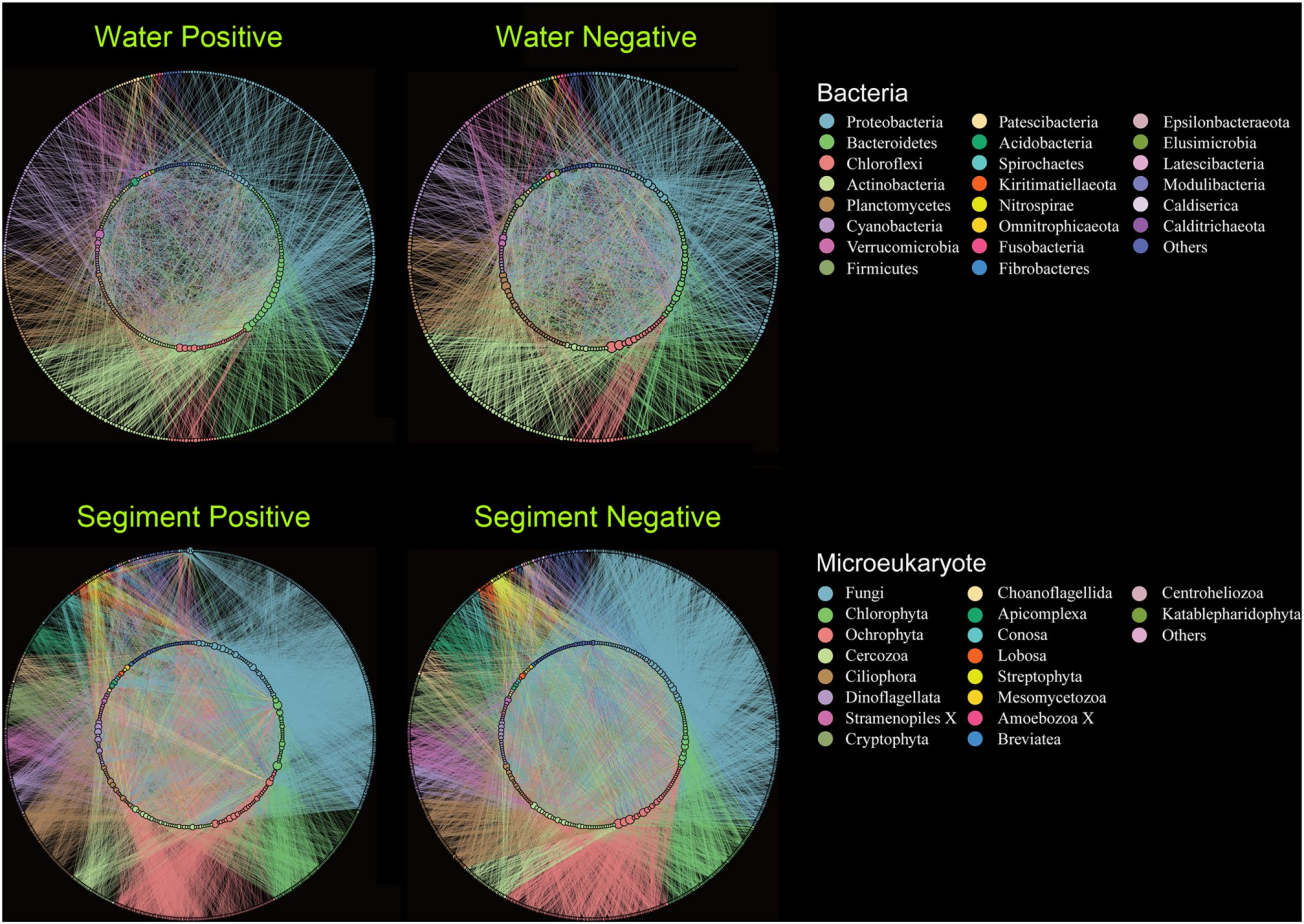
Microeukaryotic–bacterial interspecific bipartite networks. **A** Positive co-occurrence relationships in water. **B** Negative co-occurrence relationships in water. **C** Positive co-occurrence relationships in sediment. **D** Negative co-occurrence relationships in sediment. Each of the networks was constructed by the 48 samples collected from aquaculture ponds. The outer and inner circles represent bacteria and microeukaryotes, respectively. All the nodes are colored in a clockwise direction according to the legend information. The node size is proportional to links number. For the bacteria, phyla with nodes < 5 and degrees < 20 were classified as “others”. For the microeukaryotes, phyla with nodes < 5 and degrees < 10 were classified as “others”

To investigate co-occurrence bipartite networks between bacteria and microeukaryotes in coastal aquaculture ecosystems, the networks between microeukaryotes and bacteria were classified as potential positive (P) or negative (N) and interactions in water (W) or sediment (S), respectively (Fig. 1). The node number of microeukaryotes was similar in water and sediment, but bacterial nodes in sediment were about twice as abundant as in water (Table 1). Similarly, the degree in sediment was about 3.5 times higher than that in water regardless of positive or negative interactions (Table 1). In addition, the linkage density, Shannon diversity, interaction evenness and cluster coefficient all indicated that microeukaryotic–bacterial interactions in sediment were much more complex than those in water (Table 1). The low connectivity showed that only 3–5% of possible links were detected as microeukaryotic–bacterial interactions (Table 1). The microeukaryotic nodes were mainly classified to Chlorophyta, Ochrophyta, Fungi, Cercozoa, Ciliophora, Dinoflagellata, and Cryptophyta. The bacterial nodes were mainly classified to Proteobacteria, Bacteroidetes, Chloroflexi, Actinobacteria, Planctomycetes, Cyanobacteria, Verrucomicrobia, and Firmicutes (Table 2). For the positive bipartite networks in water, Chlorophyta showed the highest degree (42%), followed by Ochrophyta (13%) and Fungi (11%). For the negative bipartite network in water, Chlorophyta also had the highest degree (25%), followed by Ochrophyta (17%), Ciliophora (16%) and Fungi (13%). In both the positive and negative bipartite networks in sediment, Fungi had the highest degree, followed by Ochrophyta, Chlorophyta, Cercozoa and Dinoflagellata (Table 2). In water, most microeukaryotes showed equal weights between the node abundance and degree while a few did not. For example, Chlorophyta had a percentage of 24% in the microeukaryotic nodes, which accounted for 42% of degrees, whereas Cercozoa, Ciliophora and Dinoflagellata had fewer degrees compared to their node percentage in the positive bipartite network of water (Table 2). Also, Ochrophyta had a percentage of 10% in the total nodes but accounted for 17% of degrees in the negative bipartite network of water (Table 2). Moreover, the nested overlap and decreasing fill (NODF) that represented the nestedness was about twice as high in sediment than in water. Compared to the random network, the bipartite networks in water had no significant nestedness but the positive and negative networks in sediment showed obvious nestedness (Supplementary Fig. S1, *P* < 0.05).

**Table 1:** Overview of the topological parameters in bipartite networks between bacteria and microeukaryotes in water and sediment

Parameter	Water_positive	Water_negative	Sediment_positive	Sediment_negative
Nodes of microeukaryotes	200	164	210	208
Nodes of bacteria	408	352	761	722
Links per species	4.04	4.39	8.75	8.74
Degree	2454	2265	8501	8127
Connectance	0.03	0.04	0.05	0.05
NODF	8.9	10.8	20.4	22.1
Module number	10	29	6	8
Number of compartment	20	12	9	6
linkage density	25.6	25.0	64.0	65.7
Shannon diversity	7.8	7.7	9.0	9.0
Interaction evenness	0.69	0.70	0.75	0.75
Cluster coefficient of microeukaryotes	0.09	0.10	0.14	0.15
Cluster coefficient of bacteria	0.06	0.08	0.11	0.11

Each bipartite network was constructed with 1210 (water) and 3604 (sediment) bacterial OTUs; 406 (water) and 557 (sediment) microeukaryotic OTUs in the 48 samples collected from aquaculture ponds. NODF, nested overlap and decreasing fill

**Table 2:** Overview of degree and node percentages in the bipartite network between bacteria and microeukaryotes in water and sediment

Phylum	Water_positive		Water_negative		Sediment_positive		Sediment_negative	
	Degree%	Node%	Degree%	Node%	Degree%	Node%	Degree%	Node%
Bacteria								
Proteobacteria	34.1	34.3	33.7	32.4	38.0	31.1	40.8	32.5
Bacteroidetes	11.6	13.5	12.3	14.8	9.7	10.8	9.0	10.2
Chloroflexi	4.3	4.2	5.3	4.5	15.2	14.3	15.4	14.4
Actinobacteria	20.9	14.0	22.0	15.6	4.2	4.2	4.0	4.3
Planctomycetes	8.8	8.3	10.1	8.5	6.5	7.1	7.0	7.2
Cyanobacteria	10.1	14.0	7.1	10.8	3.0	3.3	2.9	3.0
Verrucomicrobia	4.3	3.7	3.4	5.1	3.3	4.3	3.3	3.7
Firmicutes	1.1	1.2	1.1	1.1	4.8	4.1	3.6	4.3
Patescibacteria	2.0	2.7	2.0	2.3	1.6	2.8	1.4	2.5
Acidobacteria	0	0.2	0.1	0.6	2.0	3.2	2.5	3.7
Spirochaetes	0.2	0.2	0	0.3	2.2	3.7	1.6	3.0
Epsilonbacteraeota	0	0.2	/	/	1.2	0.5	1.3	0.6
Kiritimatiellaeota	/	/	/	/	1.4	1.6	1.3	1.4
Nitrospirae	/	/	/	/	2.2	1.1	2.5	1.2
Microeukaryotes								
Fungi	11.0	13.5	12.9	15.2	24.5	20.0	21.9	20.2
Chlorophyta	41.8	23.5	24.9	20.7	15.3	12.4	15.4	10.6
Ochrophyta	12.9	12.5	16.6	9.8	16.0	14.3	20.7	13.9
Cercozoa	3.9	8.0	5.7	7.9	12.0	15.7	13.8	15.9
Ciliophora	7.2	14.5	16.3	17.7	8.2	8.6	7.4	8.7
Dinoflagellata	0.5	2.5	1.1	2.4	10.2	7.1	7.1	7.7
Stramenopiles X	8.5	5.0	5.1	4.9	3.5	5.2	3.6	4.3
Cryptophyta	5.2	8.0	7.8	7.9	0.1	0.5	0.1	0.5
Choanoflagellida	0.4	1.5	0.1	1.2	0.6	1.0	0.5	1.4
Apicomplexa	3.2	1.5	1.5	1.2	2.5	1.0	1.8	1.0
Conosa	0.1	1.0	0.5	1.2	0.7	1.0	0.7	1.0
Lobosa	/	/	/	/	1.3	1.4	2.0	1.4
Centroheliozoa	1.1	0.5	1.7	0.6	/	/	/	/

Each bipartite networks was constructed with 1210 (water) and 3604 (sediment) bacterial OTUs; 406 (water) and 557 (sediment) microeukaryotic OTUs in the 48 samples collected from aquaculture ponds. Only the phyla with degree or node percentage > 1% in any of the four bipartite networks were presented

### Degree distribution and specialization

**Fig. 2:**
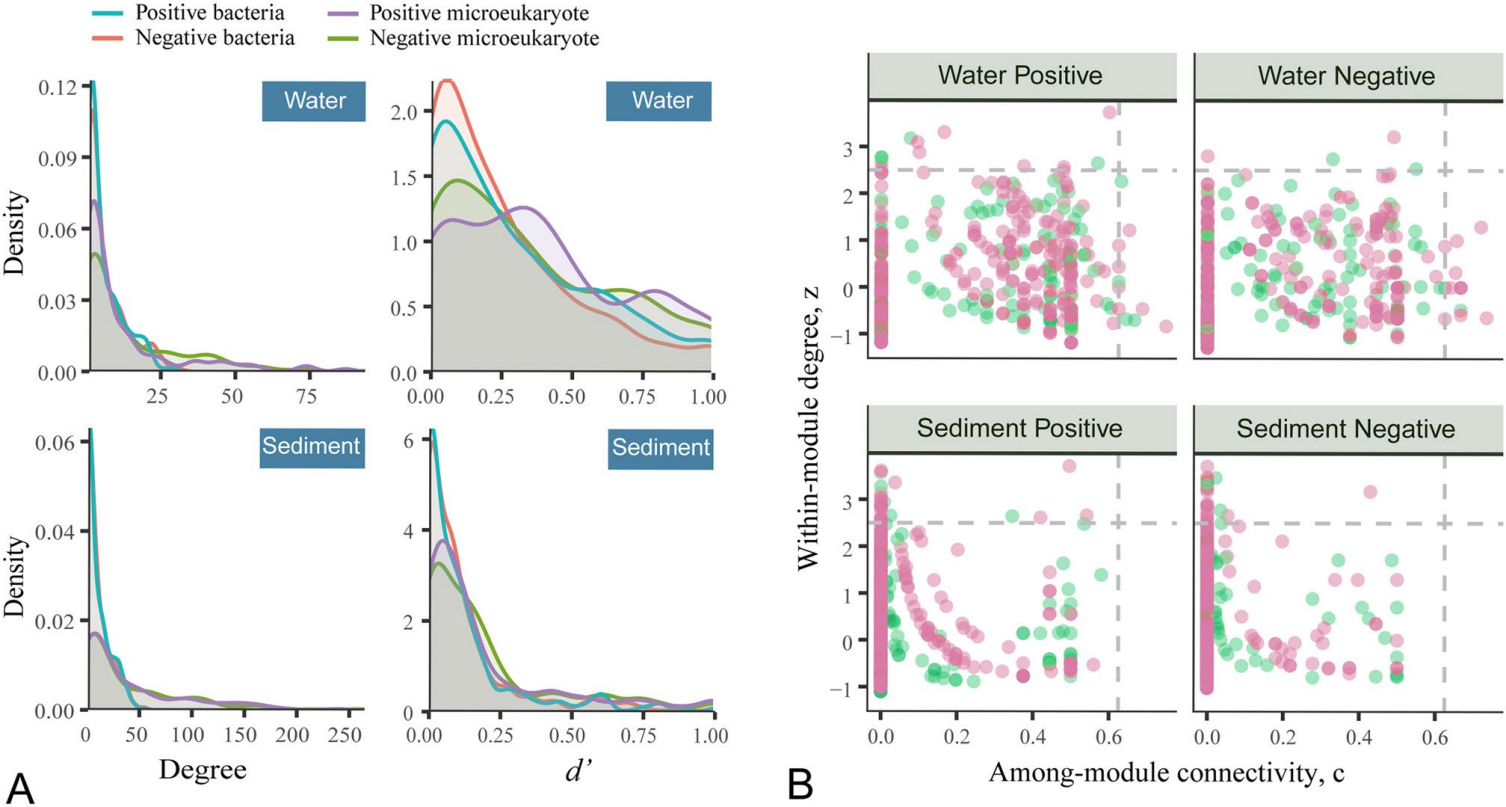
Density plot and potential keystone taxa for the positive and negative bipartite networks in water and sediment. **A** Number of links/edges/interactions and specialization index (*d*.^*’*^) between microeukaryotes and bacteria. The specialization index (*d’*) ranges from 0 (generalist) to 1 (specialist). **B** Among-module connectivity (c) and within-module connectivity (z) values for bacteria (red) and microeukaryotes (green) in each network. Dashed grey lines indicated critical values for identifying keystones according to Olesen et al. (2007)

In order to evaluate the degree distribution pattern of microeukaryotes and bacteria, the density plot for degree and specialization indices was analysed (Fig. 2A). The majority of microeukaryotes and bacteria had low degrees (Fig. 2A). The node number of microeukaryotes and bacteria decreased dramatically when the degrees reached 25 and 50 in water and sediment, respectively (Fig. 2A). In water, the largest degree of bacteria and microeukaryotes was 31 and 95, respectively. In sediment, the largest degree of bacteria and microeukaryotes was 57 and 267, respectively. In both water and sediment, the specialization index (*d’*) was close to 0, indicating that most microeukaryotes and bacteria tended to be generalists (Fig. 2A). According to the *d’* = 1, the number of pairing partners in the positive and negative bipartite networks of water were 20 and 11, respectively (Supplementary Tables S1, S2). The specific pair partners in the positive and negative bipartite networks of sediment were 6 and 4, respectively (Supplementary Tables S2, S3).

### Core microeukaryotes in the bipartite network

**Fig. 3:**
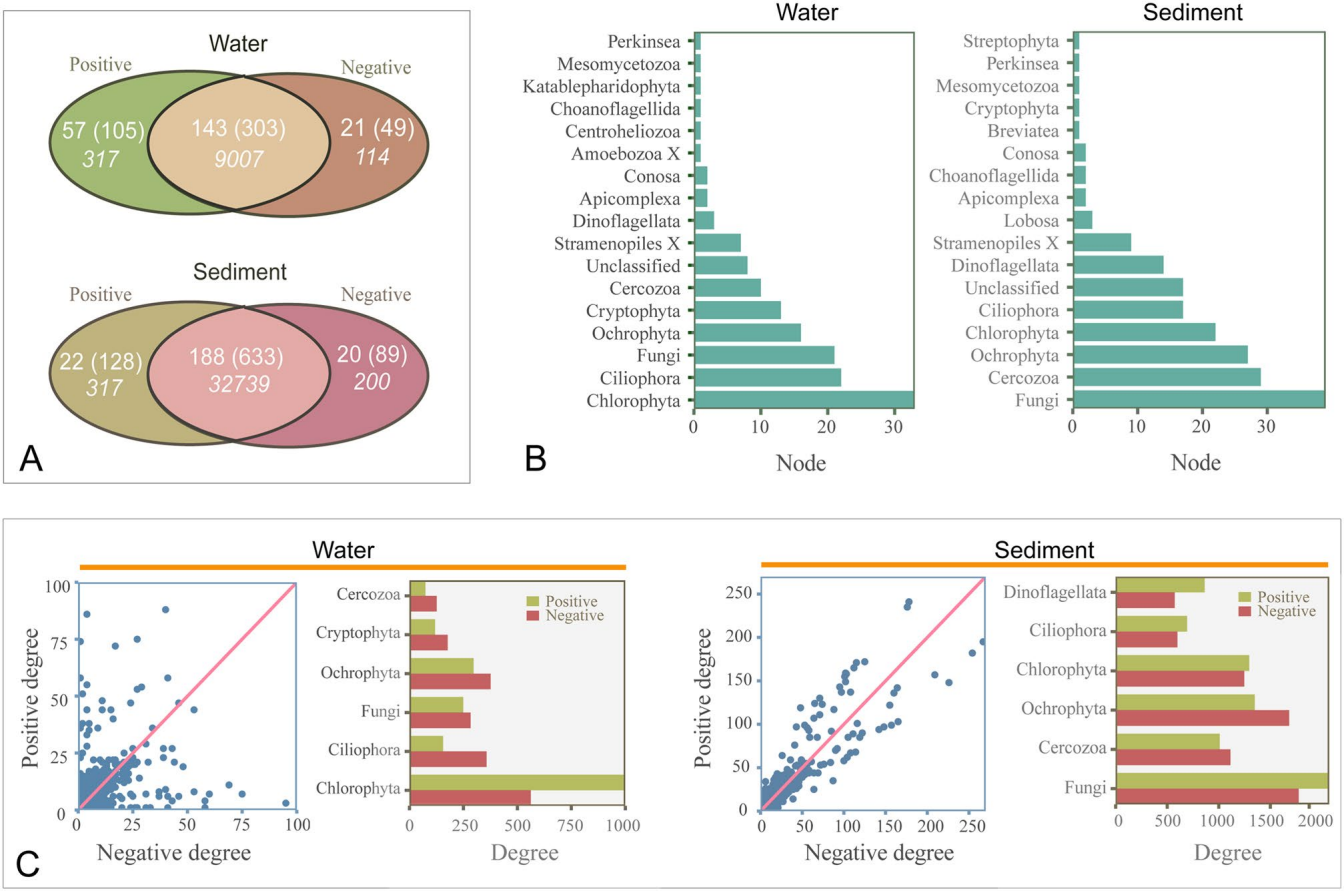
Core microeukaryotes and their degree distributions. **A** Core microeukaryotes that had both positive and negative relationships with bacteria; Numbers of microeukaryotic and bacterial nodes were given outside and inside of parentheses, respectively. The italic numbers represented the links between microeukaryotes and bacteria. **B** Node numbers of the core microeukaryotes at the phylum level. **C** Positive and negative relationships between core microeukaryotes and bacteria. Positive and negative degree numbers of top 6 core microeukaryotic phyla

In order to detect core microeukaryotes in the bipartite network between microeukaryotes and bacteria, the Venn diagram between positive and negative bipartite networks in water and sediment, was further compared (Fig. 3). Core microeukaryotes were defined as those that had both positive and negative links with bacteria in the bipartite network. In water, it was found that 143 microeukaryotes and 303 bacteria had both positive and negative relationships, which accounted for 95% of the total degrees (Fig. 3A) and they were mainly from Chlorophyta, Ciliophora, Fungi, Ochrophyta, Cryptophyta, Cercozoa, and Dinoflagellata (Fig. 3B). In sediment, 188 microeukaryotes and 633 bacteria shared the positive and negative relationships, which accounted for 98% of the total degrees (Fig. 3A) and they were mainly from Fungi, Cercozoa, Ochrophyta, Chlorophyta, Ciliophora, Dinoflagellata and Lobosa (Fig. 3B). The positive and negative degree patterns were different between water and sediment. In water, some of the microeukaryotes had biased positive or negative degrees, especially the microeukaryotes, which exhibited large degrees (Fig. 2C, Supplementary Tables S5, S6). However, most microeukaryotes had balanced positive and negative degrees in sediment (Fig. 3C). A similar pattern was also detected at the phylum level (Fig. 3C). For example, Chlorophyta had more positive degrees compared to negative degrees, while Ciliophora had more negative degrees than positive degrees in water (Fig. 3C). In sediment, most eukaryotes had equal positive and negative degrees at the phylum level (Fig. 3C). Furthermore, it was found that most nodes that had more positive degrees than negative degrees belonged to Chlorophyta and Ochrophyta (Supplementary Table S5). Also, most nodes that had more negative degrees than positive degrees belonged to Ochrophyta, Ciliophora and Cercozoa (Supplementary Table S6).

The positive and negative degrees between core microeukaryotes and bacteria were further investigated at the phylum level (Supplementary Fig. S2). In water, it was found that only Chlorophyta had more negative relationships with Cyanobacteria, whereas Fungi, Ochrophyta, Ciliophora, Cryptophyta, and Cercozoa had more positive relationships with Cyanobacteria (Supplementary Fig. S2). Ciliophora, Cryptophyta and Cercozoa had more negative relationships with Proteobacteria, Actinobacteria and Bacteroidetes (Supplementary Fig. S2). In sediment, Fungi, Chlorophyta, Cercozoa, Ciliophora and Dinoflagellata generally had equal positive and negative links with bacteria at the phylum level (Supplementary Fig. S2).

### Keystone taxa associated with network modularity

To explore keystone taxa associated with identified network modules, the within-module connectivity (*z*) and among-module connectivity (*c*) was calculated. Four microeukaryotes and 12 bacteria were detected as module connectors, which play essential roles among modules in the microeukaryotic–bacterial bipartite network of water (Fig. 2B). Fifteen microeukaryotes and 34 bacteria were detected as module hubs, which might play critical roles within the module in both water and sediment (Fig. 2B). Three of the four microeukaryotic connectors were found to belong to Ciliophora (Supplementary Table S3). Also, most module hubs of microeukaryotic connectors belonged to Chlorophyta (4), Ochrophyta (4) and Fungi (4) (Supplementary Table S4). For the bacterial module connectors, Proteobacteria (5) was the dominant phylum and there was a species belonging to Nitrosomonadaceae (Supplementary Table S7). For the bacterial module hubs, species mainly belonged to Proteobacteria (8), Planctomycetes (4), Cyanobacteria (5), Firmicutes (4), Bacteroidetes (3) and Chloroflexi (3) (Supplementary Table S4). Among the Proteobacteria, a methylotroph OM43 clade belonging to Methylophilaceae was detected (Table S8). Also, a sulfate-reducing bacteria (Desulfobacteraceae) was detected (Supplementary Table S8).

### Robustness of microeukaryotic–bacterial mutualistic relationships

To test the stability of potential mutualistic relationships to species loss in the positive bipartite network, cumulative extinctions following simulated species loss was examined (Supplementary Fig. S3). The mutualistic relationships were more robust with random extinction of partners in sediment than those in water for both microeukaryotes (0.83 vs. 0.75) and bacteria (0.90 vs. 0.78, Supplementary Fig. S3). In contrast, selective elimination starting with generalists promoted the generation of secondary extinctions for both microeukaryotes and bacteria in water and sediment (Supplementary Fig. S3). In this situation, removal of microeukaryotic generalists (0.42) promoted the secondary loss compared to bacterial generalists (0.63). However, there was no difference between water and sediment when the microeukaryotic or bacterial generalists were lost first (Supplementary Fig. S3).

## Discussion

Potential microeukaryote-bacteria interactions in water and sediment of coastal aquaculture ponds were identified by bipartite networks. Chlorophyta and fungi were identified as the dominant taxa in the microeukaryotic–bacterial bipartite networks in the water and sediment, respectively. Some microeukaryotes with high degrees had asymmetric links with bacteria in water, while microeukaryotes tended to have symmetric links with bacteria in sediment. The mutualistic relationships in sediment had a better nestedness than in water, which might contribute to its robustness following simulated random species extinctions. Although the loss of microeukaryotic and bacterial generalists could lead to the collapse of potential mutualistic relationships, the loss of microeukaryotic generalists was more threatening than that of bacterial generalists to the ecosystem stability.

Chlorophyta–bacteria interactions accounted for most positive predicted interactions in the plankton community. Generally, the ecological relationships between algae and bacteria represent the most important inter-kingdom associations in aquatic environments (Seymour et al. 2017). First, Chlorophyta provides O_2_ through photosynthesis for bacterial consumption (Zhang et al. 2020). Bacteria can then directly obtain carbon from dissolved organic carbon and algal products released by Chlorophyta (Seymour et al. 2017). Furthermore, green algae and bacteria will often form mutualistic relationships. For example, bacteria can provide B-vitamins for the algae in exchange for fixed carbon (Cooper et al. 2019). Therefore, Chlorophyta-bacteria predicted interactions dominated the potential microbial cross-kingdom interactions in water. In sediment, it was found that fungi had a dominant role in the microeukaryotic–bacterial bipartite network. Fungi also played a major role in the microbial network interactions of intertidal mangrove sediment (Marie Booth et al. 2019). However, the role of the interactions between fungi and other microorganisms is underrepresented (Bjorbækmo et al. 2020). In sediment, fungi are frequently dominant in organic matter degradation hotspots, such as in marine snow (Bochdansky et al. 2017). Moreover, fungi play a central role in the degradation of recalcitrant organic matter, such as lignocellulose (Boer et al. 2005). The fungi hyphae further increase their interaction opportunities with the bacteria (Warmink et al. 2011). Fungi and bacteria are not always in competition with each other but contribute to organic matter degradation in a mutualistic relationship (Frey-Klett et al. 2011).

The keystone taxa are generally regarded as the taxa that have a high impact on the structure and functioning of the ecosystem. A mounting number of studies define keystone taxa as those that are highly connected and centrally clustered in a microbial network (Banerjee et al. 2018; Berry and Widder 2014). Some connectors and module hubs were also identified as potential keystone taxa for maintaining the microeukaryotic–bacterial bipartite network. Three out of four identified microeukaryotic connectors belonged to Ciliophora were found, indicating that ciliates play important roles among the module connections. In microbial food webs, ciliates mainly feed on bacteria which can be influenced by the size, motility, shape and cell surface characteristics of bacteria (Hahn and Hofle 2001). Furthermore, some bacteria are resistant to digestion by ciliates (Gong et al. 2016). Thus, selective predation by ciliates on bacteria may influence the microeukaryotic–bacterial interactions. Ciliates can also predate on algae to control plankton community diversity (Beaver and Crisman 1989; Rosetta and McManus 2003). It was found that some microeukaryotes had asymmetric positive and negative links with bacteria, indicating that microeukaryotic species with asymmetric connections to bacteria may be critical in maintaining the stability of the plankton community. Thus, the variations of these microeukaryotes will amplify unbalanced disturbances between microeukaryotes and bacteria.

It was found that the loss of microeukaryotes and generalists would promote the collapse of microeukaryotic–bacterial co-occurrence relationships. The algae–bacteria positive interactions may not require microbial cells to have physical contact. For instance, the phycosphere, enriched in organic molecules (Seymour et al. 2017), can provide broadcast cues for the free-living bacteria (Seymour et al. 2017). Some bacteria can directly attach to the algal cell and utilize the substrates on the algal cell surface (Enke et al. 2018). Also, fungi and bacteria usually live together and have close spatial relationships with bacteria as epiphytes on fungi (Deveau et al. 2018). In addition, fungi can efficiently utilize phytoplankton organic matter and transfer the phytoplankton-derived organic matter to the microbial loop (Senga et al. 2018). Therefore, the loss of algae or fungi will lead to the collapse of microeukaryotic–bacterial mutualistic relationships. Microeukaryotic–bacterial mutualistic relationships in water were more vulnerable than those in sediment to disturbances with random species loss. One explanation is that sediment has a higher protistan and bacterial diversity than the water column, which may promote microbial community stability (Girvan et al. 2005; Zheng et al. 2017b, 2021). Microeukaryotic–bacterial relationships in sediment had significantly more nestedness than that in water column (Burgos et al. 2007).

How the microeukaryotic–bacterial co-occurrence network could guide microbial bioremediations was further explored. Generally, most exogenous microorganisms bring mutualistic and antagonistic impacts to the native microorganisms. Previous study has indicated that supplementation of bacterial consortia failed to improve the water quality but facilitated blue-green algae blooms (Zheng et al. 2017c). Similarly, protist predation was found to inhibit the denitrification effectiveness of denitrifying bacteria in a pilot-scale bioreactor (Ikeda-Ohtsubo et al. 2013). Thus, neglecting microeukaryotic–bacterial interactions may lead to a failure in microbial remediation. In addition, it was found that microeukaryotes and generalists were more important than bacteria and specialists in maintaining potential microbial mutualistic relationships. It was also found that the dominant taxa (e.g., Chlorophyta and Fungi) in the microeukaryotic–bacterial bipartite network played critical roles in maintaining the stability of bacterial communities. Phytoplankton successions that drive the bacterial community structure and assembly were frequently observed in the natural water (Durham et al. 2019; Kimbrel et al. 2019; Zhou et al. 2019). Yang et al. (2020) found that manipulating the chlorophytes resulted in a more stable bacterioplankton community. Here, we highlight the observation that the dominant microeukaryotic generalists, identified from the potential mutualistic interactions, might be key factors in regulating bacterioplankton communities. Furthermore, some microeukaryotes with asymmetric positive or negative interactions with bacteria can be used to facilitate or depress the growth of bacteria when necessary.

## Conclusions

This study showed that sediment of aquaculture pond ecosystems have more intensive microeukaryotic–bacterial interactions than those in water. Chlorophyta and fungi were dominant taxa in predicted microeukaryotic–bacterial interactions in water and sediment, respectively. Most microeukaryotes had symmetric positive and negative links with bacteria in both water and sediment. Moreover, most microeukaryotes and bacteria tended to be generalists, and only a few potential interactions were specialized. The microeukaryotic–bacterial mutualistic relationships in sediment were more robust than that in water, and the loss of microeukaryotic generalists will lead to the quick collapse of the mutualistic relationships. This study provides basic knowledge of microeukaryotic–bacterial cross-kingdom relationships in coastal aquaculture ecosystems, which may guide further microbial remediation in aquaculture and other aquatic ecosystems.

## Materials and methods

### Experimental design and sampling

Water and sediment samples were collected from coastal aquaculture ponds, which cultured different sizes (larval, small juvenile, large juvenile, adult fish) of grass carp. These ponds, which had an area of 1.5 km^2^ and a depth of 3 m, were located at the Pearl River Estuary (22°35′5.26′′N, 113°37′56.23′′E). Detailed environmental information of these ponds can be found in Zheng et al. (2021). For each size of the cultured grass carp, three individual ponds were selected as replicates and there were four sampling points in each pond. At each sampling point, 1 L surface water (50 cm below water level) and 500 g of surface sediment (0–10 cm) were collected. In total, 48 water samples and 48 sediment samples were collected between April and May 2018. All the collected samples were kept in a portable fridge (4 °C) and transported to the laboratory within 1 h. For each water sample, 500 ml of water was filtered through a membrane filter (0.22 μm pore size, Whatman, Maidstone, UK) to collect microbial cells. The filters and sediments were stored at − 80 °C for further DNA extraction.

### DNA isolation, PCR amplification, and sequencing

Total DNA of microbes in water collected on the filters was extracted using a PowerWater DNA Isolation Kit (MoBio Laboratories, Carlsbad, USA). The total DNA of microbes in sediment was extracted by the freeze-grind method followed by PowerSoil DNA Isolation Kit (MoBio Laboratories, Carlsbad, USA). Primers TAReuk454FWD1 (5'-CCAGCASCYGCGGTAATTCC-3') and TAReukREV3 (5'-ACTTTCGTTCTTGATYRA-3') were used to amplify the V4 region of the eukaryotic 18S rRNA gene (Stoeck et al. 2010). The universal primers 515F (5′-GTGYCAGCMGCCGCGGTAA-3') and 806R (5′-GGACTACHVGGGTWTCTAAT-3') were used to target the V4 region of the bacterial 16S rRNA gene (Caporaso et al. 2011). The microeukaryotic and bacterial rRNA genes were amplified according to the PCR programs, following previous studies (Wang et al. 2021; Zheng et al. 2021). Equimolar amounts of the purified PCR products of each sample were combined and sequenced using an Illumina HiSeq platform 2500 (Illumina, Inc., San Diego, CA, USA) with a 2 × 250 bp kit by the Biomarker Technologies Corporation (Beijing, China).

### Sequences analysis

The raw reads were mapped to sample barcodes, and primer sequences were trimmed with one nucleotide mismatch. Low quality bases were removed. Btrim was then used to remove those reads with an average quality thread of 20 at a window size of five. The FLASH program (Magoc and Salzberg 2011) were used to merge the paired end sequences (> 200 bases) with at least 10 bp overlap. Only the sequences without any ambiguous bases and > 250 bp were kept for forwarding analysis. Then, chimeras were removed against the Silva database, and sequences were clustered at 97% identity into operational taxonomy units (OTUs) by UPARSE and singletons were discarded (Edgar 2013). The 18S rRNA and 16S rRNA gene sequences were assigned to microeukaryotic and bacterial taxa by the ribosomal database project (RDP) classifier (Wang et al. 2007), based on Protist Ribosomal Reference (PR^2^) database 4.11.0 (Guillou et al. 2013) and SILVA 132 database (Quast et al. 2012), respectively. Sequences assigned to Metazoa and Archaea were excluded from subsequent analysis. The microeukaryotic and bacterial sequences were rarefied to 31,781 and 49,706 sequences per sample, respectively. All analyses were performed on a public available Galaxy pipeline (http://mem.rcees.ac.cn:8080/) (Feng et al. 2017).

### Bipartite network construction and analysis

The co-occurrence bipartite networks between microeukaryotes and bacteria were constructed using high-throughput sequencing data based on both abundance and occurrence information. Co-occurrence networks have OTU as nodes and OTU-OTU pairs as edges, where an edge may imply a biologically or biochemically meaningful relationship between OTUs (Weiss et al. 2016). In order to construct a reliable co-occurrence network, only OTUs that were detected at least in half of the 48 samples were kept. Thus, a total of 1210 and 3604 bacterial OTUs, 406 and 557 microeukaryotic OTUs were kept from the water and sediment samples, respectively. The sparse correlations for compositional data (SparCC) (Friedman and Alm 2012) were used to construct the co-occurrence network. SparCC iteratively estimates the linear Pearson correlations between the log transformed relative abundances of each OTU. SparCC is better suited to avoid spurious correlations at the cost of higher computational complexity. To make a robust network, the threshold values for constructing the networks were set at a correlation coefficient > 0.6 and *P* < 0.05 according to a previous study (Feng et al. 2019). Then, the interspecies correlations between microeukaryotes and bacteria were extracted from the whole network and a bipartite network analysis was performed to explore the potential interactions between microeukaryotes and bacteria using Bipartite 2.15 (Carsten et al. 2009) in R 4.0.1 (R Core Team 2010). The bipartite networks were visualized using Gephi (0.9.1).

The network modularity for each network was characterized by the algorithm of Beckett (2016). The connectivity of each node was quantified by its within-module connectivity (*z*) and among-module connectivity (*c*) (Olesen et al. 2007). Specifically, node topologies were classified into module hubs (highly connected nodes within modules, *z* > 2.5 and *c* ≤ 0.62), network hubs (highly connected nodes within the entire network, *z* > 2.5 and *c* > 0.62), connectors (nodes that connect modules, *z* ≤ 2.5 and *c* > 0.62) and peripherals (nodes connected in modules with few outside connections, *z* ≤ 2.5 and *c* ≤ 0.62) (Olesen et al. 2007).

Species loss refers to the microorganism that became inactive in the co-occurrence microbial network. For example, microorganism can enter a dormant state or exit interaction networks because of low abundance. The robustness of the networks to cumulative loss of single nodes was tested following Burgos et al. (2007). In brief, species loss was simulated by cumulatively and randomly removing nodes from the network. When another node on the other side of the network was connected only to the removed nodes, it was also removed from the network (secondary loss). Two situations of species loss were simulated. First, the nodes were removed from generalists to specialists using selective extinctions. This procedure was then repeated with random elimination of microeukaryotes or bacteria. A hundred randomizations were run for each network. The area below each curve (R) was used as a robustness index to cumulative node losses (Burgos et al. 2007). The value ‘1’ indicates a network highly robust to secondary species loss and value ‘0’ corresponds to networks that have already collapsed after the first few nodes were removed.

The nested overlap and decreasing fill (NODF) index were used to measure the degree of nestedness of each network. The values of nestedness range from 0 (a completely disordered network) to 100 (a perfectly nested network). The significance of NODF was estimated with a Monte Carlo procedure with 100 randomizations by Patefield's *r2dtable* algorithm (Patefield 1981). We calculated the *P* value as the proportion of random matrices that had a higher or lower index than the value observed matrix.

## Supplementary Information

Below is the link to the electronic supplementary material.

Supplementary file1 (DOCX 819 kb)

## Data Availability

Raw sequence data have been deposited in the NCBI database under BioProject accession numbers PRJNA629611 and PRJNA579535.
