# Supplementary material for: Deciphering microeukaryotic–bacterial co-occurrence networks in coastal aquaculture ponds
Source: Mar Life Sci Technol. 2023 Feb 22;5(1):44–55. doi: 10.1007/s42995-022-00159-6 (PMC10077187; doi:10.1007/s42995-022-00159-6)
Supplement: Supplementary file 1 — Supplementary file1 (DOCX 819 kb) [file 42995_2022_159_MOESM1_ESM.docx]

**Deciphering** **microeukaryotic–bacterial** **co-occurrence networks in** **coastal aquaculture ponds**

**Xiafei Zheng^a, b^, Kui Xu^b, c^,** **Jonathan Naoum^d^, Yingli Lian^b, e^, Bo Wu^b^, Zhili He^b^, Qingyun Yan^b *^**

^a^ Ninghai Institute of Mariculture Breeding and Seed Industry, Zhejiang Wanli University, Ningbo 315100, China

^b^ Environmental Microbiomics Research Center, School of Environmental Science and Engineering, Southern Marine Science and Engineering Guangdong Laboratory (Zhuhai), State Key Laboratory for Biocontrol, Sun Yat-sen University, Guangzhou 510006, China

^c^ Hubei Key Laboratory of Edible Wild Plants Conservation and Utilization, Hubei Engineering Research Center of Special Wild Vegetables Breeding and Comprehensive Utilization Technology, College of Life Sciences, Hubei Normal University, Huangshi 435002, China

^d^ Department of Biological Sciences, GRIL - EcotoQ - TOXEN, Ecotoxicology of Aquatic Microorganisms Laboratory, Université Du Québec à Montréal, Succursale Centre-Ville, Montreal, Quebec, Canada

^e^ Animal Husbandry and Fisheries Research Center of Guangdong Haid Group CO., Ltd. Key Laboratory of Microecological Resources and Utilization in Breeding Industry, Ministry of Agriculture and Rural Affairs, Guangzhou, 510006, China

Corresponding author: Qingyun Yan, E-mail: [yanqingyun@sml-zhuhai.cn](mailto:yanqingyun@sml-zhuhai.cn)

Table S1 Taxonomy and classification of the 20 specific partners in the positive bipartite network between microeukaryotes and bacteria in water.

| Partners | Domain | Phylum | Class | Order | Family | Genus | Species |
| --- | --- | --- | --- | --- | --- | --- | --- |
| Partner1 | Microeukaryote | Cercozoa | Endomyxa | Vampyrellida | Leptophryidae | Leptophryidae X | Leptophryidae X sp. |
| Partner1 | Bacteria | Proteobacteria | Unclassified | Unclassified | Unclassified | Unclassified | Unclassified |
| Partner2 | Microeukaryote | Chlorophyta | Trebouxiophyceae | Chlorellales | Chlorellales X | Nannochloris | Nannochloris sp. |
| Partner2 | Bacteria | Proteobacteria | Alphaproteobacteria | Rickettsiales | Mitochondria | Picochlorum sp. SENEW3 | Picochlorum sp. SENEW3 |
| Partner3 | Microeukaryote | Chlorophyta | Chlorophyceae | Chlamydomonadales | Chlamydomonadales X | Unclassified | Unclassified |
| Partner3 | Bacteria | Proteobacteria | Gammaproteobacteria | Gammaproteobacteria Incertae Sedis | Unknown Family | Candidatus Ovatusbacter | uncultured bacterium |
| Partner4 | Microeukaryote | Chlorophyta | Chlorophyceae | Sphaeropleales | Sphaeropleales X | Radiococcus | Radiococcus polycoccus |
| Partner4 | Bacteria | Unclassified | Unclassified | Unclassified | Unclassified | Unclassified | Unclassified |
| Partner5 | Microeukaryote | Chlorophyta | Chlorophyceae | Chlamydomonadales | Chlamydomonadales X | Chloromonas | Chloromonas kasaiae |
| Partner5 | Bacteria | Bacteroidetes | Ignavibacteria | OPB56 | Candidatus Kapabacteria sp. 59-99 | Candidatus Kapabacteria sp. 59-99 | Candidatus Kapabacteria sp. 59-99 |
| Partner6 | Microeukaryote | Chlorophyta | Trebouxiophyceae | Chlorellales | Chlorellales X | Nannochloris | Nannochloris sp. |
| Partner6 | Bacteria | Cyanobacteria | Oxyphotobacteria | Chloroplast | Unclassified | Unclassified | Unclassified |
| Partner7 | Microeukaryote | Ciliophora | CONTH 3 | CONTH 3 X | CONTH 3 XX | CONTH 3 XXX | CONTH 3 XXX sp. |
| Partner7 | Bacteria | Cyanobacteria | Oxyphotobacteria | Chloroplast | Lepocinclis sp. Psurononuma100609I | Lepocinclis sp. Psurononuma100609I | Lepocinclis sp. Psurononuma100609I |
| Partner8 | Microeukaryote | Ciliophora | Heterotrichea | Heterotrichea X | Condylostomatidae | Linostomella | Linostomella sp. |
| Partner8 | Bacteria | Proteobacteria | Alphaproteobacteria | Holosporales | Holosporaceae | Candidatus Bealeia | uncultured bacterium |
| Partner9 | Microeukaryote | Ciliophora | Phyllopharyngea | Cyrtophoria 4 | Chilodonellidae | Chilodonellidae X | Chilodonellidae X sp. |
| Partner9 | Bacteria | Actinobacteria | Actinobacteria | PeM15 | metagenome | metagenome | metagenome |
| Partner10 | Microeukaryote | Ciliophora | CONThreeP | CONThreeP X | Urotrichidae | Urotricha | Urotricha sp. |
| Partner10 | Bacteria | Proteobacteria | Alphaproteobacteria | Sphingomonadales | Sphingomonadaceae | uncultured | Unclassified |
| Partner11 | Microeukaryote | Ciliophora | Colpodea | Colpodea X | Cyrtolophosidida | Cyrtolophosis | Cyrtolophosis sp. |
| Partner11 | Bacteria | Bacteroidetes | Bacteroidia | Bacteroidetes VC2.1 Bac22 | uncultured Bacteroidetes bacterium | uncultured Bacteroidetes bacterium | uncultured Bacteroidetes bacterium |
| Partner12 | Microeukaryote | Conosa | Variosea | Variosea X | Phalansteriidae | Phalansterium | Phalansterium sp. |
| Partner12 | Bacteria | Bacteroidetes | Bacteroidia | Bacteroidetes VC2.1 Bac22 | uncultured Bacteroidetes bacterium | uncultured Bacteroidetes bacterium | uncultured Bacteroidetes bacterium |
| Partner13 | Microeukaryote | Dinoflagellata | Dinophyceae | Peridiniales | Kryptoperidiniaceae | Unruhdinium | Unruhdinium penardii |
| Partner13 | Bacteria | Proteobacteria | Alphaproteobacteria | Rickettsiales | Rickettsiaceae | Candidatus Megaira | uncultured bacterium |
| Partner14 | Microeukaryote | Fungi | Unclassified | Unclassified | Unclassified | Unclassified | Unclassified |
| Partner14 | Bacteria | Verrucomicrobia | Verrucomicrobiae | uncultured | uncultured bacterium | uncultured bacterium | uncultured bacterium |
| Partner15 | Microeukaryote | Fungi | Unclassified | Unclassified | Unclassified | Unclassified | Unclassified |
| Partner15 | Bacteria | Cyanobacteria | Oxyphotobacteria | Chloroplast | Unclassified | Unclassified | Unclassified |
| Partner16 | Microeukaryote | Fungi | Unclassified | Unclassified | Unclassified | Unclassified | Unclassified |
| Partner16 | Bacteria | Cyanobacteria | Oxyphotobacteria | Chloroplast | Unclassified | Unclassified | Unclassified |
| Partner17 | Microeukaryote | Ochrophyta | Bacillariophyta | Bacillariophyta X | Polar-centric-Mediophyceae | Unclassified | Unclassified |
| Partner17 | Bacteria | Cyanobacteria | Oxyphotobacteria | Chloroplast | Unclassified | Unclassified | Unclassified |
| Partner18 | Microeukaryote | Unclassified | Unclassified | Unclassified | Unclassified | Unclassified | Unclassified |
| Partner18 | Bacteria | Cyanobacteria | Oxyphotobacteria | Chloroplast | uncultured Phacus | uncultured Phacus | uncultured Phacus |
| Partner19 | Microeukaryote | Unclassified | Unclassified | Unclassified | Unclassified | Unclassified | Unclassified |
| Partner19 | Bacteria | Bacteroidetes | Bacteroidia | Flavobacteriales | Crocinitomicaceae | Fluviicola | uncultured bacterium |
| Partner20 | Microeukaryote | Unclassified | Unclassified | Unclassified | Unclassified | Unclassified | Unclassified |
| Partner20 | Bacteria | Patescibacteria | Gracilibacteria | Candidatus Peregrinibacteria | uncultured bacterium | uncultured bacterium | uncultured bacterium |

Table S2 Taxonomy and classification of the 11 specific partners in the negative bipartite network between microeukaryotes and bacteria in water.

| Partners | Domain | Phylum | Class | Order | Family | Genus | Species |
| --- | --- | --- | --- | --- | --- | --- | --- |
| Partner1 | Microeukaryote | Unclassified | Unclassified | Unclassified | Unclassified | Unclassified | Unclassified |
| Partner1 | Bacteria | Actinobacteria | Acidimicrobiia | Microtrichales | uncultured | Unclassified | Unclassified |
| Partner2 | Microeukaryote | Fungi | Unclassified | Unclassified | Unclassified | Unclassified | Unclassified |
| Partner2 | Bacteria | Bacteroidetes | Bacteroidia | Sphingobacteriales | NS11-12 marine group | uncultured Bacteroidetes bacterium | uncultured Bacteroidetes bacterium |
| Partner3 | Microeukaryote | Cercozoa | Filosa-Thecofilosea | Cryomonadida | Protaspa-lineage | Protaspa-lineage X | Protaspa-lineage X sp. |
| Partner3 | Bacteria | Bacteroidetes | Bacteroidia | Sphingobacteriales | env.OPS 17 | uncultured Bacteroidetes bacterium | uncultured Bacteroidetes bacterium |
| Partner4 | Microeukaryote | Ciliophora | Colpodea | Colpodea X | Cyrtolophosidida | Cyrtolophosis | Cyrtolophosis sp. |
| Partner4 | Bacteria | Chlamydiae | Chlamydiae | Chlamydiales | Simkaniaceae | Candidatus Renichlamydia | uncultured Candidatus Rhabdochlamydia sp. |
| Partner5 | Microeukaryote | Chlorophyta | Chlorophyceae | Sphaeropleales | Sphaeropleales X | Ankyra | Ankyra judayi |
| Partner5 | Bacteria | Cyanobacteria | Oxyphotobacteria | Synechococcales | Cyanobiaceae | Cyanobium PCC-6307 | uncultured bacterium |
| Partner6 | Microeukaryote | Cercozoa | Endomyxa | Vampyrellida | Vampyrellidae | Vampyrella | Vampyrella sp. |
| Partner6 | Bacteria | Cyanobacteria | Oxyphotobacteria | Synechococcales | Cyanobiaceae | Cyanobium PCC-6307 | uncultured bacterium |
| Partner7 | Microeukaryote | Ochrophyta | Eustigmatophyceae | Eustigmatophyceae X | Eustigmatophyceae XX | Monodus | Monodus sp. |
| Partner7 | Bacteria | Cyanobacteria | Oxyphotobacteria | Chloroplast | Unclassified | Unclassified | Unclassified |
| Partner8 | Microeukaryote | Chytridiomycota | Chytridiomycotina | Chytridiomycetes | Unclassified | Unclassified |  |
| Partner8 | Bacteria | Proteobacteria | Deltaproteobacteria | Unclassified | Unclassified | Unclassified | Unclassified |
| Partner9 | Microeukaryote | Ciliophora | Litostomatea | Litostomatea X | Litostomatea XX | Litostomatea XXX | Litostomatea XXX sp. |
| Partner9 | Bacteria | Proteobacteria | Gammaproteobacteria | Legionellales | Legionellaceae | Legionella | uncultured Legionellales bacterium |
| Partner10 | Microeukaryote | Chlorophyta | Trebouxiophyceae | Trebouxiophyceae X | Trebouxiophyceae XX | Lemmermannia | Lemmermannia tetrapedia |
| Partner10 | Bacteria | Proteobacteria | Gammaproteobacteria | Alteromonadales | Alteromonadaceae | Rheinheimera | uncultured Rheinheimera sp. |
| Partner11 | Microeukaryote | Fungi | Unclassified | Unclassified | Unclassified | Unclassified | Unclassified |
| Partner11 | Bacteria | Verrucomicrobia | Verrucomicrobiae | Opitutales | Opitutaceae | Opitutus | metagenome |

Table S3 Taxonomy and classification of the 6 specific partners in the positive bipartite network between microeukaryotes and bacteria in sediment.

| Partners | Domain | Phylum | Class | Order | Family | Genus | Species |
| --- | --- | --- | --- | --- | --- | --- | --- |
| Partner1 | Microeukaryote | Chlorophyta | Chlorophyceae | Sphaeropleales | Sphaeropleales X | Scenedesmus | Scenedesmus armatus |
| Partner1 | Bacteria | Acidobacteria | Thermoanaerobaculia | Thermoanaerobaculales | Thermoanaerobaculaceae | Subgroup 10 | bacterium enrichment culture clone Anammox_2 |
| Partner2 | Microeukaryote | Unclassified | Unclassified | Unclassified | Unclassified | Unclassified |  |
| Partner2 | Bacteria | Proteobacteria | Alphaproteobacteria | Sphingomonadales | Sphingomonadaceae | Novosphingobium | uncultured bacterium |
| Partner3 | Microeukaryote | Ciliophora | Litostomatea | Litostomatea X | Litostomatea XX | Unclassified | Unclassified |
| Partner3 | Bacteria | Chloroflexi | Anaerolineae | SBR1031 | A4b | Unclassified | Unclassified |
| Partner4 | Microeukaryote | Chlorophyta | Chlorophyceae | Sphaeropleales | Sphaeropleales X | Pediastrum | Pediastrum duplex |
| Partner4 | Bacteria | Chloroflexi | Anaerolineae | Anaerolineales | Anaerolineaceae | uncultured | metagenome |
| Partner5 | Microeukaryote | Chlorophyta | Trebouxiophyceae | Chlorellales | Chlorellales X | Dictyosphaerium | Dictyosphaerium lacustre |
| Partner5 | Bacteria | Cyanobacteria | Oxyphotobacteria | Chloroplast | Unclassified | Unclassified | Unclassified |
| Partner6 | Microeukaryote | Ochrophyta | Bacillariophyta | Bacillariophyta X | Polar-centric-Mediophyceae | Cyclotella | Cyclotella atomus |
| Partner6 | Bacteria | Omnitrophicaeota | Omnitrophia | Omnitrophales | Omnitrophaceae | Candidatus Omnitrophus | uncultured prokaryote |

Table S4 Taxonomy and classification of the 4 specific partners in the negative bipartite network between microeukaryotes and bacteria in sediment.

| Partners | Domain | Phylum | Class | Order | Family | Genus | Species |
| --- | --- | --- | --- | --- | --- | --- | --- |
| Partner1 | Microeukaryote | Fungi | Cryptomycota | Cryptomycotina | Cryptomycotina X | Cryptomycotina XX | Cryptomycotina XX sp. |
| Partner1 | Bacteria | Chloroflexi | Anaerolineae | SBR1031 | A4b | Unclassified | Unclassified |
| Partner2 | Microeukaryote | Streptophyta | Zygnemophyceae | Zygnemophyceae X | Zygnemophyceae XX | Closterium | Unclassified |
| Partner2 | Bacteria | Cyanobacteria | Oxyphotobacteria | Synechococcales | Cyanobiaceae | Cyanobium PCC-6307 | Unclassified |
| Partner3 | Microeukaryote | Lobosa | Tubulinea | Echinamoebida | Vermamoebidae | Hartmannella | Hartmannella vermiformis |
| Partner3 | Bacteria | Proteobacteria | Gammaproteobacteria | Betaproteobacteriales | Nitrosomonadaceae | mle1-7 | uncultured bacterium |
| Partner4 | Microeukaryote | Ciliophora | Litostomatea | Haptoria 4 | Didiniidae | Didiniidae X | Didiniidae X sp. |
| Partner4 | Bacteria | Proteobacteria | Deltaproteobacteria | Myxococcales | Unclassified | Unclassified | Unclassified |

Table S5 Species with asymmetric linkages that have more positive links than negative links with bacteria in water.

| OTU ID | Pos | Neg | Domain | Phylum | Class | Order | Family | Genus | Species |
| --- | --- | --- | --- | --- | --- | --- | --- | --- | --- |
| OTU_3446_M | 86 | 4 | Stramenopiles | Stramenopiles X | Oomycota | Oomycota X | Peronosporales | Peronosporales X | Peronosporales X sp. |
| OTU_3562_M | 74 | 1 | Archaeplastida | Chlorophyta | Chlorophyceae | Sphaeropleales | Sphaeropleales X | Unclassified | Unclassified |
| OTU_56_M | 74 | 1 | Archaeplastida | Chlorophyta | Chlorophyceae | Sphaeropleales | Sphaeropleales X | Mychonastes | Mychonastes afer |
| OTU_3606_M | 44 | 4 | Archaeplastida | Chlorophyta | Chlorophyceae | Sphaeropleales | Sphaeropleales X | Desmodesmus | Desmodesmus communis |
| OTU_234_M | 15 | 1 | Archaeplastida | Chlorophyta | Chlorophyceae | Sphaeropleales | Sphaeropleales X | Scenedesmus | Unclassified |
| OTU_1113_M | 15 | 2 | Archaeplastida | Chlorophyta | Chlorophyceae | Chlamydomonadales | Chlamydomonadales X | Spermatozopsis | Spermatozopsis similis |
| OTU_143_M | 58 | 1 | Archaeplastida | Chlorophyta | Trebouxiophyceae | Chlorellales | Chlorellales X | Oocystis | Oocystis lacustris |
| OTU_24_M | 14 | 1 | Archaeplastida | Chlorophyta | Trebouxiophyceae | Chlorellales | Chlorellales X | Dictyosphaerium | Dictyosphaerium lacustre |
| OTU_324_M | 36 | 1 | Opisthokonta | Fungi | Chytridiomycota | Chytridiomycotina | Chytridiomycotina X | Chytridiomycotina XX | Chytridiomycotina XX sp. |
| OTU_367_M | 33 | 4 | Opisthokonta | Fungi | Chytridiomycota | Chytridiomycotina | Chytridiomycetes | Rhizophydium | Rhizophydium chlorogonii |
| OTU_116_M | 35 | 5 | Alveolata | Ciliophora | Oligohymenophorea | Peritrichia | Sessilida | Vorticella | Vorticella convallaria |
| OTU_314_M | 55 | 4 | Stramenopiles | Ochrophyta | Eustigmatophyceae | Eustigmatophyceae X | Eustigmatophyceae XX | Unclassified | Unclassified |
| OTU_617_M | 51 | 2 | Stramenopiles | Ochrophyta | Bacillariophyta | Bacillariophyta X | Polar-centric-Mediophyceae | Cyclotella | Cyclotella striata |
| OTU_731_M | 38 | 2 | Stramenopiles | Ochrophyta | Bacillariophyta | Bacillariophyta X | Polar-centric-Mediophyceae | Cyclotella | Cyclotella atomus |
| OTU_59_M | 28 | 4 | Hacrobia | Cryptophyta | Cryptophyceae | Cryptophyceae X | Cryptomonadales | Teleaulax | Teleaulax acuta |
| OTU_49_M | 24 | 1 | Stramenopiles | Cryptophyta | Cryptophyceae | Cryptophyceae X | Cryptomonadales | Chroomonas | Unclassified |
| OTU_76_M | 16 | 1 | Rhizaria | Cercozoa | Endomyxa | Vampyrellida | Leptophryidae | Leptophryidae X | Leptophryidae X sp. |
| OTU_12_M | 13 | 1 | Rhizaria | Cercozoa | Filosa-Thecofilosea | Cryomonadida | Protaspa-lineage | Protaspa-lineage X | Protaspa-lineage X sp. |
| OTU_157_M | 12 | 1 | Rhizaria | Cercozoa | Filosa-Sarcomonadea | Cercomonadida | Paracercomonadidae | Paracercomonas | Paracercomonas sp. |

The _M in the column of OTU ID indicates this OTU is a microeukaryote. Pos and Neg represent positive and negative interactions between microeukaryotes and bacteria, respectively.

Table S6 Species with asymmetric linkages that have more negative links than positive links with bacteria in water.

| OTU ID | Pos | Neg | Domain | Phylum | Class | Order | Family | Genus | Species |
| --- | --- | --- | --- | --- | --- | --- | --- | --- | --- |
| OTU_301_M | 1 | 41 | Alveolata | Ciliophora | Colpodea | Colpodea X | Cyrtolophosidida | Cyrtolophosis | Cyrtolophosis sp. |
| OTU_64_M | 6 | 46 | Alveolata | Ciliophora | Spirotrichea | Tintinnida | Tintinnidiidae | Tintinnidium | Tintinnidium sp. |
| OTU_155_M | 1 | 33 | Alveolata | Ciliophora | Litostomatea | Rhynchostomatia | Dileptidae | Dileptidae X | Dileptidae X sp. |
| OTU_113_M | 1 | 31 | Alveolata | Ciliophora | Spirotrichea | Tintinnida | Eutintinnidae | Eutintinnus | Eutintinnus sp. |
| OTU_168_M | 1 | 10 | Alveolata | Ciliophora | Spirotrichea | Strombidiida | Strombidiidae | Spirostrombidium | Spirostrombidium agathae |
| OTU_10_M | 3 | 95 | Stramenopiles | Ochrophyta | Bacillariophyta | Bacillariophyta X | Polar-centric-Mediophyceae | Thalassiosira | Unclassified |
| OTU_1396_M | 7 | 75 | Stramenopiles | Ochrophyta | Chrysophyceae | Chrysophyceae X | Chrysophyceae Clade-C | Chrysophyceae Clade-C X | Chrysophyceae Clade-C X sp. |
| OTU_365_M | 4 | 43 | Stramenopiles | Ochrophyta | Chrysophyceae | Chrysophyceae X | Chrysophyceae Clade C | Ochromonas | Ochromonas danica |
| OTU_1351_M | 1 | 24 | Stramenopiles | Ochrophyta | Eustigmatophyceae | Eustigmatophyceae X | Eustigmatophyceae XX | Eustigmatophyceae XXX | Eustigmatophyceae XXX sp. |
| OTU_3150_M | 1 | 20 | Stramenopiles | Ochrophyta | Bacillariophyta | Bacillariophyta X | Raphid-pennate | Nitzschia | Unclassified |
| OTU_2928_M | 8 | 53 | Stramenopiles | Ochrophyta | Bacillariophyta | Bacillariophyta X | Polar-centric-Mediophyceae | Cyclotella | Cyclotella scaldensis |
| OTU_54_M | 7 | 60 | Hacrobia | Cryptophyta | Cryptophyceae | Cryptophyceae X | Cryptomonadales | Cryptomonas | Cryptomonas tetrapyrenoidosa |
| OTU_47_M | 6 | 41 | Archaeplastida | Chlorophyta | Trebouxiophyceae | Trebouxiophyceae X | Trebouxiophyceae XX | Lemmermannia | Lemmermannia tetrapedia |
| OTU_25_M | 9 | 40 | Rhizaria | Cercozoa | Endomyxa | Vampyrellida | Leptophryidae | Leptophryidae X | Leptophryidae X sp. |
| OTU_9_M | 1 | 27 | Rhizaria | Cercozoa | Endomyxa | Vampyrellida | Leptophryidae | Leptophryidae X | Leptophryidae X sp. |

The _M in the column of OTU ID indicates this OTU is a microeukaryote. Pos and Neg represent positive and negative interactions between microeukaryotes and bacteria, respectively.

Table S7 The potential keystone taxa acting as connectors among the different modules in water.

| OTU ID | Network | Domain | Phylum | Class | Order | Family | Genus | Species |
| --- | --- | --- | --- | --- | --- | --- | --- | --- |
| OTU_219_B | WP | Bacteria | Proteobacteria | Alphaproteobacteria | Rhizobiales | A0839 | uncultured bacterium | uncultured bacterium |
| OTU_16246_B | WP | Bacteria | Cyanobacteria | Oxyphotobacteria | Synechococcales | Cyanobiaceae | Cyanobium PCC-6307 | uncultured bacterium |
| OTU_29_B | WP | Bacteria | Proteobacteria | Gammaproteobacteria | Betaproteobacteriales | Burkholderiaceae | MWH-UniP1 aquatic group | uncultured bacterium |
| OTU_45_B | WN | Bacteria | Proteobacteria | Alphaproteobacteria | Rhizobiales | Rhizobiales Incertae Sedis | uncultured | uncultured bacterium |
| OTU_604_B | WN | Bacteria | Proteobacteria | Alphaproteobacteria | Unclassified | Unclassified | Unclassified | Unclassified |
| OTU_134_B | WN | Bacteria | Proteobacteria | Gammaproteobacteria | Betaproteobacteriales | Nitrosomonadaceae | Unclassified | Unclassified |
| OTU_137_B | WN | Bacteria | Bacteroidetes | Bacteroidia | Bacteroidetes VC2.1 Bac22 | metagenome | metagenome | metagenome |
| OTU_14285_B | WN | Bacteria | Verrucomicrobia | Verrucomicrobiae | Chthoniobacterales | Chthoniobacteraceae | LD29 | uncultured bacterium |
| OTU_6742_B | WN | Bacteria | Actinobacteria | Actinobacteria | Frankiales | Sporichthyaceae | hgcI clade | uncultured actinobacterium |
| OTU_161_B | WN | Bacteria | Chloroflexi | Anaerolineae | Caldilineales | Caldilineaceae | uncultured | uncultured bacterium |
| OTU_281_B | WN | Bacteria | Planctomycetes | Phycisphaerae | Phycisphaerales | Phycisphaeraceae | CL500-3 | uncultured bacterium |
| OTU_97_B | WN | Bacteria | Planctomycetes | OM190 | uncultured bacterium | uncultured bacterium | uncultured bacterium | uncultured bacterium |
| OTU_19_M | WP | Stramenopiles | Stramenopiles X | Oomycota | Oomycota X | Peronosporales | Peronosporales X | Peronosporales X sp. |
| OTU_338_M | WP | Alveolata | Ciliophora | CONThreeP | CONThreeP X | Urotrichidae | Urotricha | Urotricha sp. |
| OTU_51_M | WP | Alveolata | Ciliophora | Nassophorea | Nassophorea X | Colpodidiidae | Colpodidium | Colpodidium caudatum |
| OTU_127_M | WN | Alveolata | Ciliophora | Prostomatea 1 | Prostomatea 1 X | Colepidae | Levicoleps | Levicoleps biwae |

The _M and _B in the column of OTU ID indicate this OTU is a microeukaryoute and bacterium, respectively. WP and WN represent positive and negative network, respectively.

Table S8 The potential keystone taxa acting as module hubs within modules in the bipartite network of water and sediment.

| OTU ID | Network | Domain | Phylum | Class | Order | Family | Genus | Species |
| --- | --- | --- | --- | --- | --- | --- | --- | --- |
| OTU_169_B | WP | Bacteria | Patescibacteria | Saccharimonadia | Saccharimonadales | uncultured candidate division WS5 bacterium | uncultured candidate division WS5 bacterium | uncultured candidate division WS5 bacterium |
| OTU_217_B | WP | Bacteria | Actinobacteria | Actinobacteria | Micrococcales | Microbacteriaceae | Aurantimicrobium | uncultured bacterium |
| OTU_281_B | WP | Bacteria | Planctomycetes | Phycisphaerae | Phycisphaerales | Phycisphaeraceae | CL500-3 | uncultured bacterium |
| OTU_305_B | WP | Bacteria | Cyanobacteria | Oxyphotobacteria | Chloroplast | Unclassified | Unclassified | Unclassified |
| OTU_14_B | WP | Bacteria | Cyanobacteria | Oxyphotobacteria | Synechococcales | Cyanobiaceae | Cyanobium PCC-6307 | uncultured cyanobacterium |
| OTU_335_B | WP | Bacteria | Chloroflexi | Anaerolineae | SBR1031 | A4b | Unclassified | Unclassified |
| OTU_68_B | WP | Bacteria | Proteobacteria | Gammaproteobacteria | Betaproteobacteriales | Methylophilaceae | OM43 clade | uncultured bacterium |
| OTU_47_M | WP | Archaeplastida | Chlorophyta | Trebouxiophyceae | Trebouxiophyceae X | Trebouxiophyceae XX | Lemmermannia | Lemmermannia tetrapedia |
| OTU_87_M | WP | Stramenopiles | Stramenopiles X | Labyrinthulea | Thraustochytriales | Thraustochytriaceae | Thraustochytriaceae X | Thraustochytriaceae X sp. |
| OTU_157_M | WP | Rhizaria | Cercozoa | Filosa-Sarcomonadea | Cercomonadida | Paracercomonadidae | Paracercomonas | Paracercomonas sp. |
| OTU_153_M | WP | Opisthokonta | Fungi | Chytridiomycota | Chytridiomycotina | Chytridiomycetes | Unclassified | Unclassified |
| OTU_192_M | WP | Opisthokonta | Fungi | Unclassified | Unclassified | Unclassified | Unclassified | Unclassified |
| OTU_71_B | WN | Bacteria | Planctomycetes | Planctomycetacia | Gemmatales | Gemmataceae | uncultured | bacterium enrichment culture clone B145(2011) |
| OTU_8625_B | WN | Bacteria | Cyanobacteria | Oxyphotobacteria | Synechococcales | Cyanobiaceae | Cyanobium PCC-6307 | uncultured bacterium |
| OTU_42_M | WN | Opisthokonta | Fungi | Unclassified | Unclassified | Unclassified | Unclassified | Unclassified |
| OTU_617_M | WN | Stramenopiles | Ochrophyta | Bacillariophyta | Bacillariophyta X | Polar-centric-Mediophyceae | Cyclotella | Cyclotella striata |
| OTU_143_B | SP, SN | Bacteria | Proteobacteria | Gammaproteobacteria | Steroidobacterales | Steroidobacteraceae | uncultured | uncultured Methylococcaceae bacterium |
| OTU_57_B,  OTU_7867_B | SP, SN | Bacteria | Proteobacteria | Deltaproteobacteria | Desulfobacterales | Desulfobacteraceae | Sva0081 sediment group | uncultured soil bacterium |
| OTU_76_B | SP, SN | Bacteria | Proteobacteria | Gammaproteobacteria | Cellvibrionales | Spongiibacteraceae | BD1-7 clade | uncultured bacterium |
| OTU_63_B | SP, SN | Bacteria | Planctomycetes | Planctomycetacia | Pirellulales | Pirellulaceae | Candidatus Anammoximicrobium | uncultured bacterium |
| OTU_15_B | SP, SN | Bacteria | Planctomycetes | Phycisphaerae | Phycisphaerales | Phycisphaeraceae | CL500-3 | uncultured marine bacterium |
| OTU_282_B | SP, SN | Bacteria | Nitrospirae | Thermodesulfovibrionia | uncultured | uncultured bacterium | uncultured bacterium | uncultured bacterium |
| OTU_11_M | SP, SN | Archaeplastida | Chlorophyta | Chlorophyceae | Sphaeropleales | Sphaeropleales X | Monactinus | Monactinus sturmii |
| OTU_56_M | SP, SN | Archaeplastida | Chlorophyta | Chlorophyceae | Sphaeropleales | Sphaeropleales X | Mychonastes | Mychonastes afer |
| OTU_79_M, OTU_10_M | SP, SN | Stramenopiles | Ochrophyta | Bacillariophyta | Bacillariophyta X | Polar-centric-Mediophyceae | Thalassiosira | Unclassified |
| OTU_13444_B | SP | Bacteria | Bacteroidetes | Bacteroidia | Bacteroidales | Bacteroidetes vadinHA17 | Unclassified | Unclassified |
| OTU_203_B | SP | Bacteria | Bacteroidetes | Bacteroidia | Sphingobacteriales | Lentimicrobiaceae | uncultured prokaryote | uncultured prokaryote |
| OTU_152_B | SN | Bacteria | Bacteroidetes | Bacteroidia | Bacteroidales | Prolixibacteraceae | uncultured | uncultured bacterium |
| OTU_179_B | SP | Bacteria | Firmicutes | Clostridia | DTU014 | uncultured Selenomonadales bacterium | uncultured Selenomonadales bacterium | uncultured Selenomonadales bacterium |
| OTU_351_B | SP | Bacteria | Firmicutes | Erysipelotrichia | Erysipelotrichales | Erysipelotrichaceae | ZOR0006 | uncultured bacterium |
| OTU_48_B | SP | Bacteria | Firmicutes | Clostridia | Clostridiales | Heliobacteriaceae | Hydrogenispora | uncultured Firmicutes bacterium |
| OTU_79_B | SP | Bacteria | Firmicutes | Clostridia | Clostridiales | Clostridiaceae 1 | Clostridium sensu stricto 1 | Clostridium perfringens |
| OTU_5291_B | SP | Bacteria | Cyanobacteria | Oxyphotobacteria | Synechococcales | Cyanobiaceae | Cyanobium PCC-6307 | uncultured bacterium |
| OTU_244_B | SP | Bacteria | Cyanobacteria | Oxyphotobacteria | Chloroplast | Unclassified | Unclassified | Unclassified |
| OTU_558_B | SP | Bacteria | Proteobacteria | Deltaproteobacteria | Desulfobacterales | Desulfobacteraceae | Unclassified | Unclassified |
| OTU_6_B | SP | Bacteria | Fusobacteria | Fusobacteriia | Fusobacteriales | Fusobacteriaceae | Cetobacterium | uncultured bacterium |
| OTU_17_M | SP | Stramenopiles | Ochrophyta | Eustigmatophyceae | Eustigmatophyceae X | Eustigmatophyceae XX | Unclassified | Unclassified |
| OTU_7_M | SP | Opisthokonta | Fungi | Cryptomycota | Cryptomycotina | Cryptomycotina X | Cryptomycotina XX | Cryptomycotina XX sp. |
| OTU_231_B | SN | Bacteria | Chloroflexi | Anaerolineae | Anaerolineales | Anaerolineaceae | Longilinea | uncultured soil bacterium |
| OTU_249_B | SN | Bacteria | Chloroflexi | Anaerolineae | Anaerolineales | Anaerolineaceae | uncultured | uncultured Chloroflexi bacterium |
| OTU_7862_B | SN | Bacteria | Chloroflexi | Anaerolineae | Anaerolineales | Anaerolineaceae | Anaerolinea | uncultured bacterium |
| OTU_662_B | SN | Bacteria | Latescibacteria | uncultured Planctomycetales bacterium | uncultured Planctomycetales bacterium | uncultured Planctomycetales bacterium | uncultured Planctomycetales bacterium | uncultured Planctomycetales bacterium |
| OTU_35_B, OTU_8449_B | SN | Bacteria | Proteobacteria | Gammaproteobacteria | Betaproteobacteriales | Burkholderiaceae | Unclassified | Unclassified |
| OTU_80_B | SN | Bacteria | Proteobacteria | Gammaproteobacteria | Betaproteobacteriales | Rhodocyclaceae | Unclassified | Unclassified |
| OTU_2119_M | SN | Stramenopiles | Ochrophyta | Eustigmatophyceae | Eustigmatophyceae X | Eustigmatophyceae XX | Trachydiscus | Trachydiscus minutus |
| OTU_234_M | SN | Archaeplastida | Chlorophyta | Chlorophyceae | Sphaeropleales | Sphaeropleales X | Scenedesmus | Unclassified |

The _M and _B in the column of OTU ID indicate this OTU is a microeukaryoute and bacterium, respectively. WP and WN represent positive and negative network in water, respectively; SP and SN represent positive and negative network in sediment, respectively.


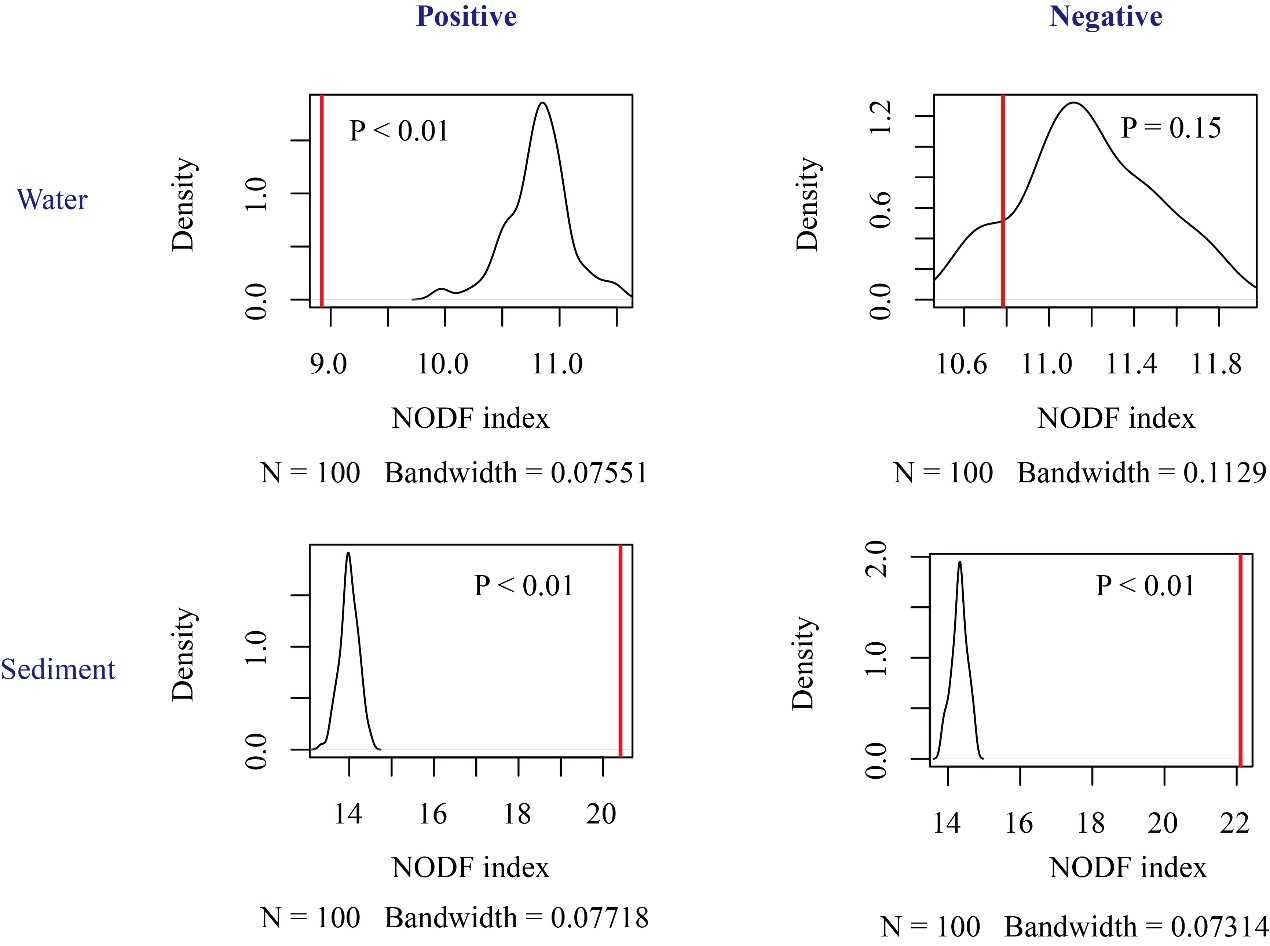


Fig. S1 Null model tests of the nested overlap and decreasing fill (NODF) between observed network and random networks in water and sediment. The larger number of NODF index indicated the network was more nested. The red line is the NODF index in each observed bipartite network. The black line represented the NODF index of 100 null models. P < 0.01 indicated the observed difference is significantly different from null model expectations.


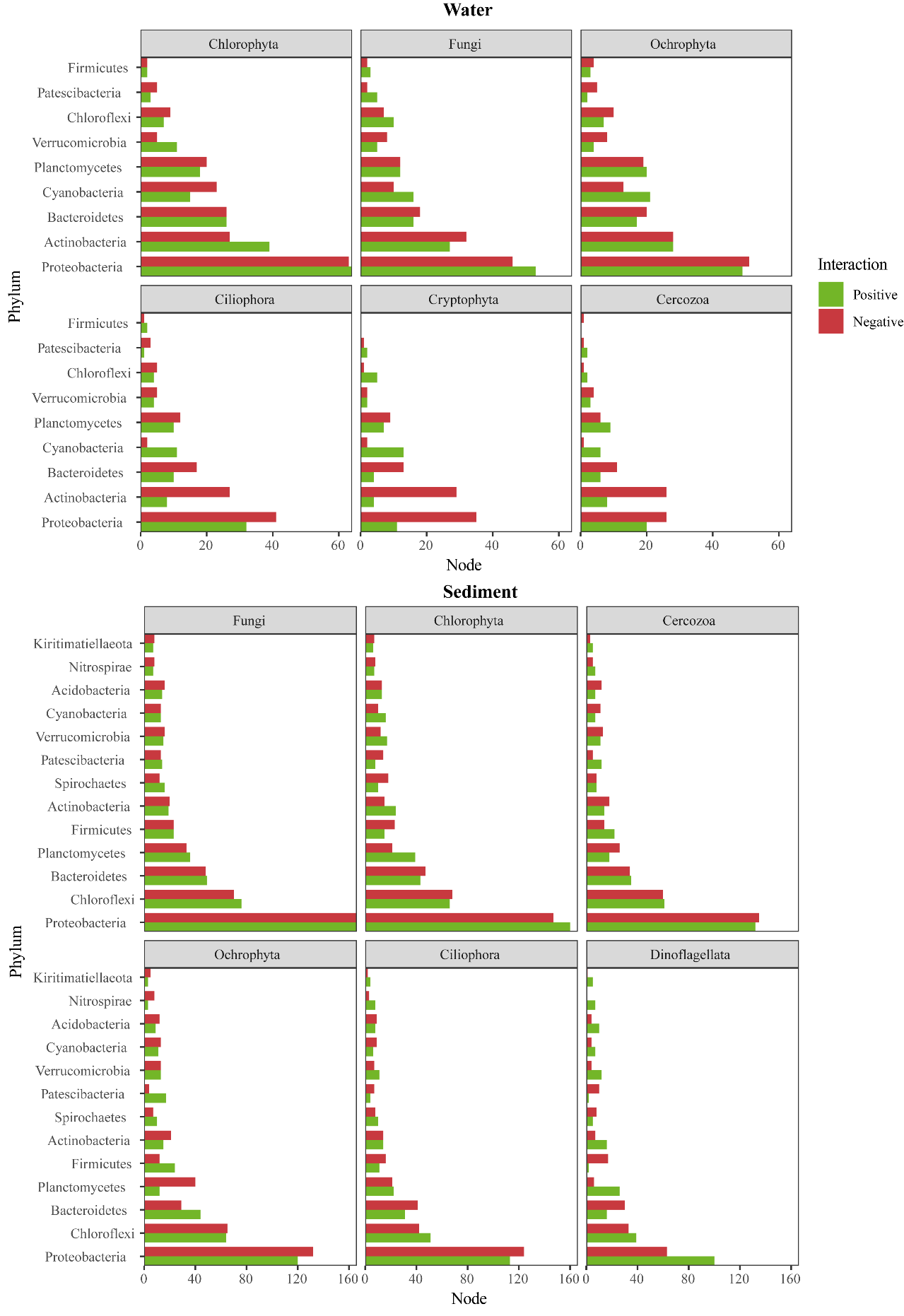


Fig. S2 Bacteria interacted with the core microeukaryotes at the phylum level. Bacterial phyla with total nodes < 5 and 10 were excluded in water and sediment, respectively.


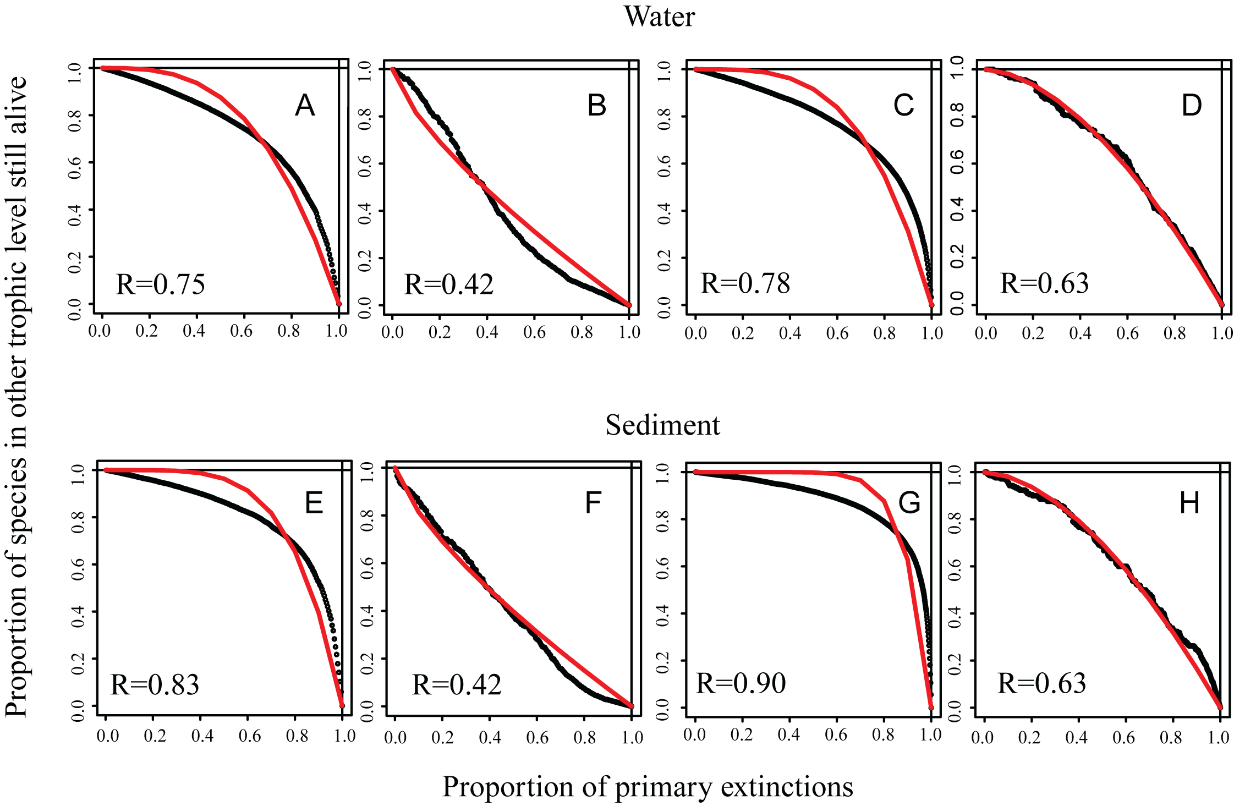


Fig. S3 Network robustness to cumulative extinctions following two different simulation procedures. (A) and (E) Random microeukaryote removal. (B) and (F) Generalist microeukaryotes were removed first. (C) and (G) Random bacteria removal. (D) and (H) Generalist bacteria were removed first. Black line represented the proportion of species that were still alive after the species in the other trophic level went extinct. The red line represented the fitted function y ~1-xa. R represented the area under the fitted curve.
